# Free Fatty Acid Palmitate Impairs the Vitality and Function of Cultured Human Bladder Smooth Muscle Cells

**DOI:** 10.1371/journal.pone.0041026

**Published:** 2012-07-13

**Authors:** Andreas Oberbach, Nadine Schlichting, Marco Heinrich, Holger Till, Jens-Uwe Stolzenburg, Jochen Neuhaus

**Affiliations:** 1 Department of Pediatric Surgery, University Hospital of Leipzig, Leipzig, Germany; 2 Leipzig University Medical Center, IFB Adiposity Diseases, Leipzig, Germany; 3 Department of Urology, University of Leipzig, Leipzig, Germany; University of Colorado, United States of America

## Abstract

**Background:**

Incidence of urinary tract infections is elevated in patients with diabetes mellitus. Those patients show increased levels of the saturated free fatty acid palmitate. As recently shown metabolic alterations induced by palmitate include production and secretion of the pro-inflammatory cytokine interleukine-6 (IL-6) in cultured human bladder smooth muscle cells (hBSMC). Here we studied the influence of palmitate on vital cell properties, for example, regulation of cell proliferation, mitochondrial enzyme activity and antioxidant capacity in hBSMC, and analyzed the involvement of major cytokine signaling pathways.

**Methodology/Principal Findings:**

HBSMC cultures were set up from bladder tissue of patients undergoing cystectomy and stimulated with palmitate. We analyzed cell proliferation, mitochondrial enzyme activity, and antioxidant capacity by ELISA and confocal immunofluorescence. In signal transduction inhibition experiments we evaluated the involvement of NF-κB, JAK/STAT, MEK1, PI3K, and JNK in major cytokine signaling pathway regulation. We found: (i) palmitate decreased cell proliferation, increased mitochondrial enzyme activity and antioxidant capacity; (ii) direct inhibition of cytokine receptor by AG490 even more strongly suppressed cell proliferation in palmitate-stimulated cells, while counteracting palmitate-induced increase of antioxidant capacity; (iii) in contrast knockdown of the STAT3 inhibitor SOCS3 increased cell proliferation and antioxidant capacity; (iv) further downstream JAK/STAT3 signaling cascade the inhibition of PI3K or JNK enhanced palmitate induced suppression of cell proliferation; (v) increase of mitochondrial enzyme activity by palmitate was enhanced by inhibition of PI3K but counteracted by inhibition of MEK1.

**Conclusions/Significance:**

Saturated free fatty acids (e.g., palmitate) cause massive alterations in vital cell functions of cultured hBSMC involving distinct major cytokine signaling pathways. Thereby, certain cytokines might counteract the palmitate-induced downregulation of cell proliferation and vitality. This could be an important link to clinical findings of increased risk of metabolic related bladder diseases such as overactive bladder (OAB) and bladder pain syndrome/interstitial cystitis (BPS/IC).

## Introduction

Abnormal urinary storage symptoms are linked to obesity and poor general health [1]. Over the past 20 years rapid increase in the number of overweight and obese individuals can be stated still increasing. Obesity related diseases are prognosed to gain increasing relevance in public health of industrialized countries. Obesity is a constellation of central adiposity and often related to impaired fasting glucose [2], elevated blood pressure [3], and dyslipidemia such as high triglyceride, low HDL cholesterol and increased levels of free fatty acid (FFA) palmitate [4].

Palmitate is an inflammatory stimulus and can induce IL-6 and MCP-1 expression and secretion in cultured human bladder smooth muscle cells [5]. Induction of cytokines by palmitate may be a pathogenetic factor underlying the higher frequency and persistence of urinary tract infections in patients with metabolic diseases [5]. Urinary tract infections (UTI) are more frequent in patients with diabetes mellitus than in subjects with normal glucose metabolism and take a more severe course [6].

Various studies have shown palmitate dependent regulation of cytokines such as IL-6 [5], [7], TNFα [8] and MCP1 [5]. Recently we reported on palmitate stimulating IL-6 abundance via NF-κB autocrine and paracrine signaling in detrusor myocytes [5]. Additionally, IL-6 is a key cytokine in cell proliferation, mitochondrial enzyme activity, antioxidant capacity [9], and intercellular communication [10].

Since basic bladder function depends on balanced cellular interactions, cytokines are a major regulation factor in normal and pathological bladder states [10]–[12]. Various cytokines are upregulated in overactive bladder [13], interstitial cystitis (IC) [14], during bladder inflammation [12], [15], and in metabolic disorders [5].

Therefore we investigated the effect of palmitate onto four major pathways involved in cytokine signaling: (1) janus kinase (JAK) activation of Signal transducers and activators of transcription (JAK/STAT); (2) Phosphoinositol-3-kinases (PI3K); (3) Mitogen-activated protein kinase kinase 1 (MEK1); (4) c-Jun N-terminal kinase (JNK).

The JAK/STAT pathway regulates vital cell processes such as cell proliferation, survival and inflammation [16]. Cell proliferation depends on phosphorylated STAT3 (pSTAT3) [17] and pSTAT3 elicits an anti-apoptotic signal via MAPK pathway [18]. Previously, we showed suppressor of cytokine signaling SOCS3 expression in hBSMC [5], which inhibits the cytokine receptor signaling via inhibition of JAK/STAT3 [19].

PI3K pathway is involved in cytokine modulation of cell proliferation, cell motility and cell survival [20]. This signaling cascade plays an important role in metabolic disease such as obesity related disorders [21].

MEK1 regulates activation of extracellular signal-regulated kinase (ERK) [22], which also influences vital cell functions such as proliferation and cell cycle arrest [23].

JNK is a mitogen-activated protein kinase, which regulates gene transcription via cJUN activation, associated with oxidative stress and inflammation [24].

In the present study we investigated palmitate effects on vital cellular functions of cultured human bladder smooth muscle cells and their modulation by inhibition of some major cytokine signaling pathways. We found that palmitate inhibited cell proliferation, and increased mitochondrial activity and antioxidant capacity. Those functions are differentially modulated by inhibition of major cytokine signaling pathways.

## Methods

### Ethics Statement

The study was approved by the Ethics Committee of the University of Leipzig (Reg. No. 773) and was conducted according to the principles expressed in the Declaration of Helsinki. Written informed consent was obtained from all patients.

### Cell Culture

Human bladder smooth muscle cell (hBSMC) cultures were established from human detrusor muscle after radical cystectomy of tumor patients as described earlier [5]. Cells were grown in SMC Growth Medium 2 (PromoCell, Heidelberg, Germany) and passages P4–P6 were used for the experiments. At 80% confluence cells were treated with 0.25 mM palmitate for 48 h to analyze palmitate effect on cell proliferation, mitochondrial enzyme activity and antioxidant capacity. A palmitate concentration of 0.25 mM, which is the postprandial blood level found in humans [25] proved to be non-toxic for hBSMC in pilot dose-finding studies (data not shown). Palmitate was used in combination with 2% bovine serum albumin (BSA) as carrier of FFAs. Medium treated cells were used as control.

For pathway analyses, cells were pre-incubated for 1 h with 40 µM MG132 (proteasome inhibitor of NF-κB signaling; TOCRIS, Ellisville, MO, USA), 2 µM AG490 (inhibitor of Janus Kinase; MERCK, Darmstadt, Germany), 20 µM PD98059 (inhibitor of Map Kinase Kinase/Erk Kinase 1 [26]; Calbiochem, San Diego, CA, USA), 20 µM LY294002 (inhibitor of Phosphatidylinositol 3-Kinase [27]; Calbiochem) and 50 µM SP600125 (inhibitor of c-Jun N-terminal Kinase [28]; TOCRIS).

### Small Interfering RNA (siRNA) Transfection

Knockdown of SOCS3 was performed by SOCS3 siRNA transfection of hBSMC. Cells were grown to 80% confluency in 6-well plates. After 24 h cells were treated with 10 nM SOCS3 siRNA (Hs_SOCS3_7_HP siRNA; Qiagen, Hilden, Germany) in serum free medium. For transfection HiPerFect Transfection Reagent (Qiagen) were used. As negative, non-silencing control the AllStars Negative Control siRNA, including in this kit, was used. The Negative Control siRNA was labeled with Alexa Fluor 488 fluorescence dye, which allowed monitoring of transfection efficiency by LSM-5 Pascal confocal laser scanning microscope (Carl Zeiss, Jena, Germany). SOCS3 knockdown efficiency was detected by quantitative PCR after 24 h of transfection.

### RNA Extraction and Quantitative PCR

Total RNA was isolated with AllPrep DNA/RNA/Protein Mini Kit (Qiagen). Quantitative PCR was performed with the real-time PCR-System realplex2 Mastercycler (Eppendorf, Hamburg, Germany) using the SYBR-Green quantitative PCR Mastermix (Fermentas, St. Leon-Rot, Germany) and custom primers (MWG-Biotech, Ebersberg, Germany; Table 1). Human 36B4 (acidic ribosomal phosphoprotein P0) served as internal standard.

**Table 1:** List of primers used for quantitative PCR.

Primer	sequence 5′→3′	length (bp)	binding site
h36B4 forward	AAC ATG CTC AAC ATC TCC CC	397	exon 6
h36B4 reverse	CCG ACT CCT CCG ACT CTT C		exon 8
hBcl-XL forward	TCT GGT CCC TTG CAG CTA GT	197	exon 3
hBcl-XL reverse	CAG GGA GGC TAA GGG GTA AG		exon 3
hPGC1α forward	GGC AGT AGA TCC TCT TCA AGA TC	262	exon 8/9
hPGC1α reverse	TCA CAC GGC GCT CTT CAA TTG		exon 10/11
hGPX1 forward	TGG CTT CTT GGA CAA TTG CG	272	exon 1
hGPX1 reverse	GAT GCC CAA ACT GGT TGC ACG GGA A		exon 1
hCAT forward	GCG GTC AAG AAC TTC ACT GA	188	exon 11/12
hCAT reverse	GCT AAG CTT CGC TGC ACA GGT		exon 13
hCOX1 forward	CAA TGC CAC CTT CAT CCG A	430	exon 4
hCOX1 reverse	GAG CCG CAG TTG ATA CTG A		exon 7
h4BP1 forward	TAT GAC CGG AAA TTC CTG ATG GA	159	exon 2
h4BP1 reverse	CCG CTT ATC TTC TGG GCT ATT G		exon 2

### Dot Blot Analysis

Protein extracts were prepared according to manufactory manual (AllPrep DNA/RNA/Protein Mini Kit; Qiagen). 2 µg total protein were transferred in triplicates on nitrocellulose membrane by Dot Blotting (Dot Blot 96 System, Biometra, Goettingen, Germany). After blocking with Odyssey blocking buffer (LICOR Biosciences, Bad Homburg, Germany) for 1 h the membranes were incubated with anti-SOCS3 rabbit IgG (1∶1000; Santa Cruz Biotechnology, Heidelberg, Germany) over night at 4°C. For detection we used anti-rabbit IRDye 680 (1∶5000; Licor Biosciences) for 2 h. Membranes were scanned with Odyssey Infrared Imager and evaluated by Odyssey Infrared Imaging Software 3.0 (Licor Biosciences). Total protein was visualized by SYPRO Ruby blot stain (BioRad, Munich, Germany).

### Assays

For cell proliferation and mitochondrial enzyme activity analysis cells were used at 80% confluency in 96-well plates and incubated over night.

Cell proliferation was measured by BrdU colorimetric cell proliferation ELISA (Roche, Mannheim, Germany) by quantifying BrdU incorporated into the newly synthesized DNA of replicating cells.

Mitochondrial enzyme activity was analyzed using 3-(4,5-Dimethylthiazol-2-yl)-2,5-diphenyltetrazolium bromide (MTT) assay. MTT (a yellow tetrazole; Sigma-Aldrich, Steinheim, Germany) is reduced to purple formazan in living cells. Hydrochloric acid is added to dissolve the insoluble purple formazan product into a colored solution. The absorbance of this colored solution can be quantified by measuring at 570 nm by a spectrophotometer.

The antioxidant capacity was measured by Antioxidant Assay (Cayman Chemical Company, Ann Arbor, MI, USA), which show the cumulative effect of all antioxidants present in cell lysate include enzymes (superoxide dismutase, catalase, glutathione peroxidase), macromolecules (albumin, ceruloplasmin, ferritin) and small molecules (ascorbic acid, alpha-tocopherol, beta-carotene, reduced gluthatione, uric acid, bilirubin).

Secreted IL-6 was quantified by Quantikine human IL-6 immunoassay (R&D Systems, Minneapolis, MN, USA). IL-6 in cell culture supernatants were measured after being centrifuged in 1∶100 diluted samples.

### Confocal Immunofluorescence

Cells were cultured on cover slips. At 80% confluence cells were fixed in 4% buffered paraformaldehyde. For detection of protein carbonylation cells were pre-incubated 1 h with 0.1% 2,4-dinitrophenyl hydrazine (DNPH; Sigma-Aldrich, Steinheim, Germany) which reacts with protein carbonyls. Cells were incubated over night at 4°C with primary antibodies (Table 2) for Dinitrophenol (DNP), a marker for protein carbonylation; Malondialdehyde (MDA), a marker for lipid oxidation, pSTAT3 and SOCS3, regulated proteins of IL-6 signaling. Indirect immunofluorescence was performed with secondary antibodies conjugated with Alexa Fluor 488 or Alexa Fluor 555 fluorescence dye (1∶500; Invitrogen, Karlsruhe, Germany). Cell nuclei were stained with 4′6- diamidino-2-phenylindoldihydro-chloride (DAPI). The cells were analyzed at a LSM-5 Pascal confocal laser scanning microscope (Carl Zeiss).

**Table 2:** List of primary antibodies used for immunocytochemistry.

Antigen	host	Type	source	dilution
human pSTAT3 (Tyr705)	mouse	monoclonal, IgG1	Cell Signaling Technology, Danvers, USA	1∶100
human SOCS3 (H-103)	rabbit	polyclonal, IgG	Santa Cruz Biotechnology, Heidelberg, Germany	1∶100
Malondialdehyde (MDA)	rabbit	polyclonal, IgG	abcam, Cambridge, UK	1∶400
Dinitrophenol (DNP)	mouse	monoclonal, IgG1	Acris Antibodies, Herford, Germany	1∶400

### Statistical Analysis

Complete data analysis was performed using Prism 5.0 (GraphPad Software, La Jolla, USA) statistical software. The data are presented as the mean +/− SEM from at least six independent experiments. Statistical differences were analyzed by ANOVA. A P-value <0.05 was considered statistically significant.

## Results

### Palmitate Effects on Cell Proliferation, Mitochondrial Enzyme Activity and Antioxidant Capacity

**Figure 1:**
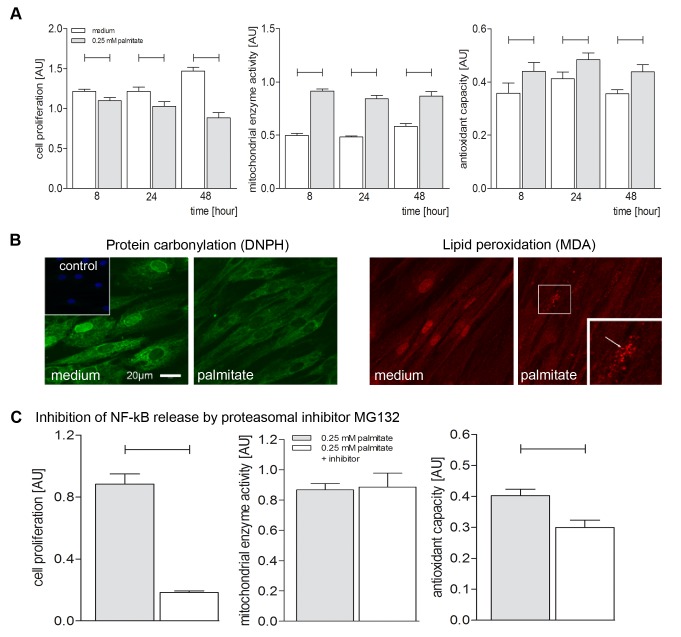
Palmitate effects on cell proliferation, mitochondrial enzyme activity and antioxidant capacity by inhibition NF-κB-signaling on human Bladder Smooth Muscle Cells. Cultured hBSMC (P4–P6) after 8, 24 and 48 h stimulation with 0.25 mM palmitate. (**A**) Time-dependent palmitate effects on cell proliferation, mitochondrial enzyme activity and antioxidant capacity measured by ELISA. (**B**) Confocal Immunofluorescence for protein carbonylation (DNPH, green) and lipid peroxidation (MDA, red) either medium control or after 48 h stimulation with 0.25 mM palmitate. Upper left inset represents negative control without primary antibody. Nuclei were stained with DAPI (blue). Lower right inset represents 2-fold magnification of peroxidated lipid droplets (white arrow). (**C**) Regulation of cell proliferation, mitochondrial enzyme activity and antioxidant capacity after previous inhibition of proteasomal degradation of IkB leading to diminished NF-κB release with 40 mM MG132 for 48 h. Data are shown as mean and SEM from three different cultures. Significant differences are indicated by bars. The medium controls are depicted in Fig. 1A and are omitted in Fig. 1C (48 h stimulation).

Treatment of hBSMC with FFA palmitate (up to 48 hrs) resulted in significant decrease of cell proliferation in a time dependent manner (Fig. 1A). Furthermore phase contrast microscopic observation indicated early signs of palmitate induced apoptosis in vital hBSMC (Fig. S1). In contrast, mitochondrial enzyme activity was significantly increased by 0.25 mM stimulation up to 48 hours (Fig. 1A). Cell stimulation with 0.25 mM palmitate showed up-regulation of antioxidant capacity, (Fig. 1A). Additionally, confocal microscopy revealed decreased protein carbonylation (Fig. 1B) compared to medium after 48 hours of 0.25 mM palmitate stimulation. On the other hand, malone dialdehyde (MDA), a marker of lipid peroxidation, showed distinct lipid oxidative droplets located to the cell membrane after 48 hours of palmitate stimulation whereas in medium control we did not observed peroxidated lipid droplets (Fig. 1B). Taken together, palmitate had a significant effect on cell proliferation, mitochondrial enzyme activity and an inverse effect on antioxidant capacity.

It is well known that palmitate induces NF-κB via protein kinase-C (PKC)-activity and that NF-κB release can be blocked by MG132, a potent inhibitor of proteasomal degradation of IkB [29]. After 48 h simultaneous stimulation with 0.25 mM palmitate + inhibitor MG132 we found significant decrease in cell proliferation and in antioxidant capacity, while there was no effect on mitochondrial enzyme activity (Fig. 1C).

**Figure 2:**
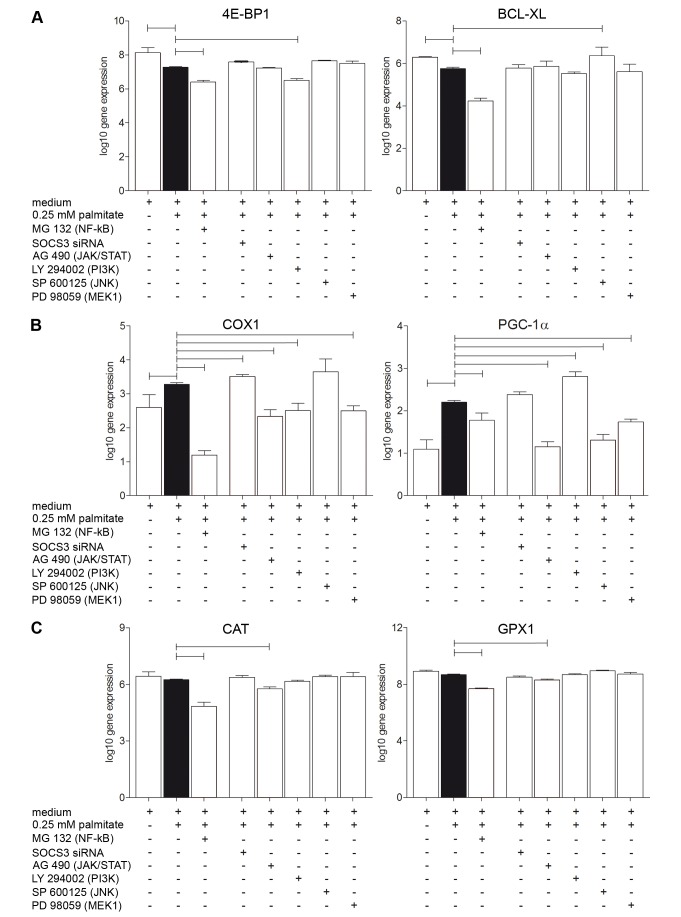
Alterations in gene expression by Palmitate and cytokine signaling inhibitors. Regulation of genes related to cell proliferation (**A**), mitochondrial enzyme activity (**B**) and antioxidant capacity (**C**) after 48 h stimulation with 0.25 mM palmitate (black column) and after simultaneous inhibition of cytokine signaling mediators. Data are shown as mean ± SEM; significant differences to palmitate are indicated by bars; p<0.05.

In gene expression experiments we found down-regulation by NF-κB of genes related to cell proliferation (4E-BP1 and BCL-XL, Fig. 2A), mitochondrial enzyme activity (COX1 and PGC-1α, Fig. 2B), and to antioxidant capacity (CAT and GPX1, Fig. 2C).

Interestingly, 4E-BP1 and BCL-XL were already down regulated by palmitate alone, while COX1 and PGC-1α were up-regulated. Palmitate had no effect on genes involved in antioxidant capacity neither CAT nor GPX1 (Fig. 2A–C).

### Palmitate Induced IL-6 Signaling Influenced Cell Proliferation, Mitochondrial Enzyme Activity and Antioxidant Capacity

In previous studies we have shown that palmitate leads to significant increase of IL-6 protein expression, cellular secretion, and IL-6 signaling. Here we investigated the influence of IL-6 pathways inhibition, prototypic for numerous other cytokines.

**Figure 3:**
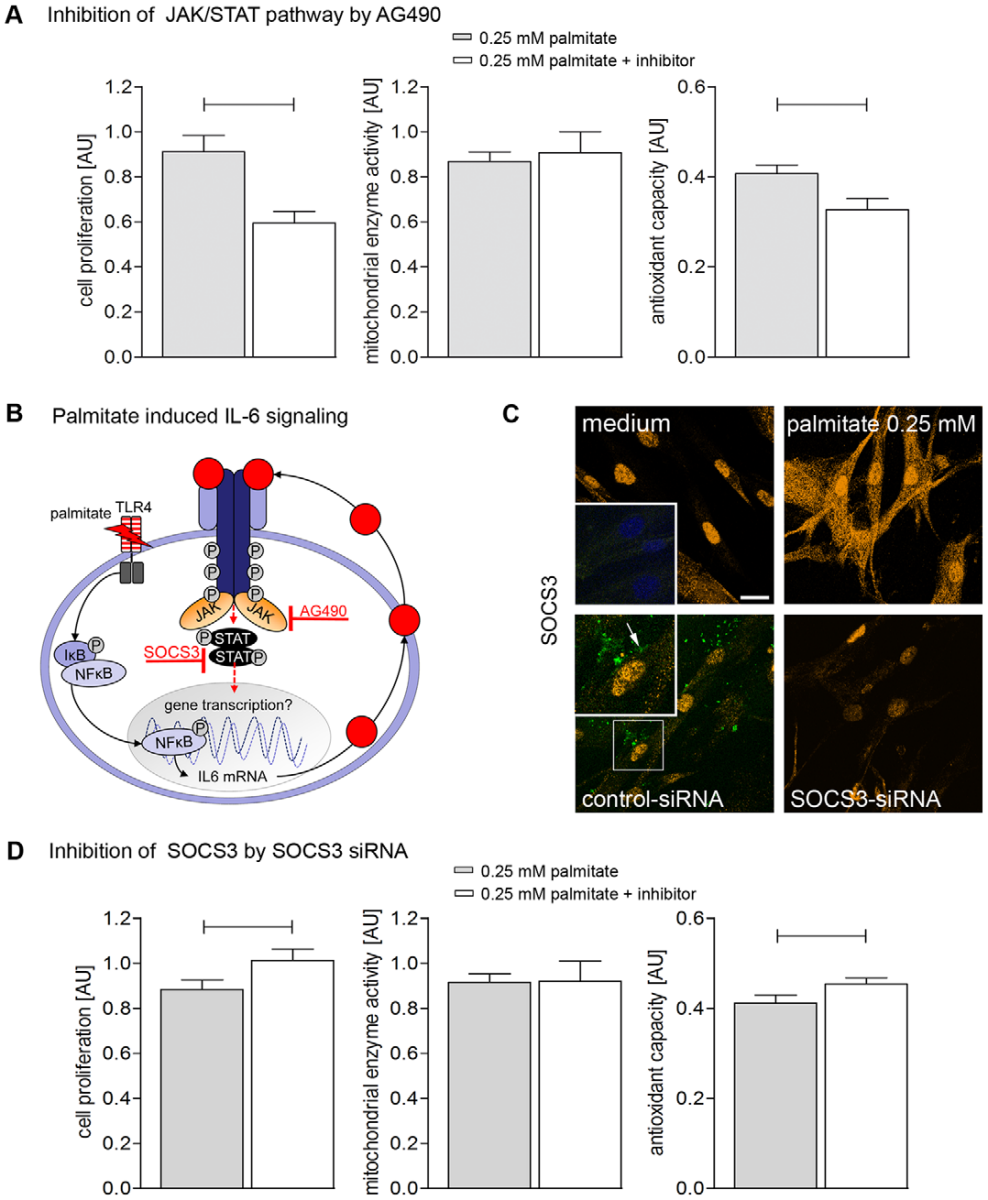
Modulation of palmitate effects on cell proliferation, mitochondrial enzyme activity and antioxidant capacity by inhibition of IL-6-JAK/STAT pathway. (**A**) Cell proliferation, mitochondrial enzyme activity and antioxidant capacity after 48 h stimulation with 0.25 mM palmitate and simultaneous inhibition of JAK/STAT signaling pathway with AG490. (**B**) Scheme of palmitate induced cytokine secretion via NF-κB activation at the example of IL6 and autocrine IL6-signaling. The cytokine receptor signaling pathway can be completely blocked by AG490 which inhibits the receptor associated Janus kinase (JAK). (**C**) Confocal immunofluorescence for SOCS3 (orange) expression in hBSMC either medium control (control), after palmitate stimulation (palmitate), co-staining with control-siRNA (green, arrow) and after SOCS3-siRNA incubation. Upper inset represents negative control without primary antibody. Nuclei were stained with DAPI (blue). Lower inset represents 2-fold magnification of same image (**D**) Cell proliferation, mitochondrial enzyme activity and antioxidant capacity after 48 h stimulation with 0.25 mM palmitate and previous SOCS3 inhibition. Appropriate medium controls are depicted in Fig. 1A (48 h stimulation). Data are shown as mean and SEM from three different cultures. Significant differences are indicated by bars. For appropriate medium controls refer to Fig. 1A (48 h stimulation).

We followed a two step approach in the present study: (i) we investigated the JAK/STAT receptor signaling using AG490 for direct inhibition of the cytokine receptor, and we used SOCS3 inhibition to release full STAT3 signaling cascade, (ii) we analyzed the signalling downstream JAK/STAT. Palmitate stimulation under direct inhibition of the cytokine receptor by the specific JAK/STAT inhibitor AG490 significantly decreased cell proliferation and antioxidant capacity while mitochondrial activity was unaltered (Fig. 3A). This was reflected by congruent downregulation of antioxidant capacity key enzyme gene expression (CAT and GPX1, Fig. 2C). While gene expression of mitochondrial enzymes COX1 and PGC-1α was downregulated by AG490 (Fig. 2B) there was no detectable effect in protein activity assay (Fig. 3A).

**Figure 4:**
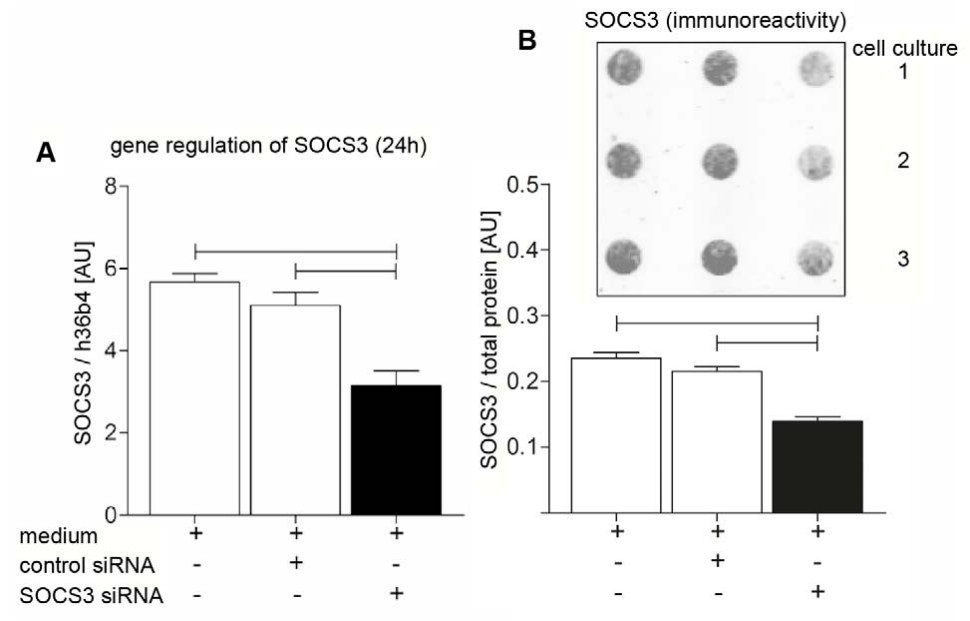
Suppression of SOCS3 on mRNA and protein level. (**A**) Inhibition of SOCS3 mRNA expression (black column) after 24 h incubation with SOCS3 siRNA in relation to reference gene h36B4. (**B**) Inhibition of SOCS3 protein expression (black column) after 24 h incubation with SOCS3 siRNA analyzed via Dot Blot after SOCS3 inhibition. Immunoreactivity of SOCS3 protein (inset) was related to total protein content stained with SYPRO-Ruby. Data are shown as mean and SEM from three different cultures. Significant differences are indicated by bars.

As illustrated in figure 3B SOCS3 regulates gene transcription by inhibition of STAT3. We performed small interfering RNA (siRNA)-mediated knockdown of SOCS3 to release full cytokine receptor signaling. We found less SOCS3-immunoreactivity in confocal analysis of SOCS3-siRNA treated cells (Fig. 3C). The effectivity of siRNA knockdown was verified by real-time PCR and protein analysis (Fig. 4). Stimulation of hBSMC with 0.25 mM palmitate + inhibition of SOCS3 led to up-regulation of cell proliferation and antioxidant capacity (Fig. 3D).

**Figure 5:**
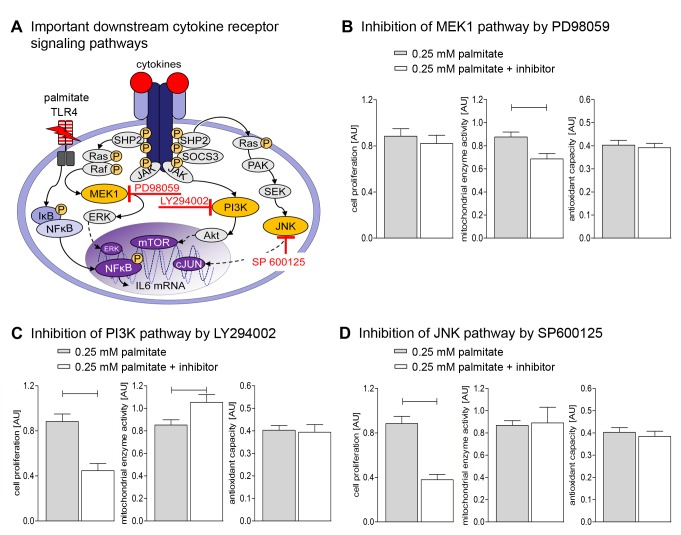
Palmitate effect on cell proliferation, mitochondrial enzyme activity and antioxidant capacity by selective inhibition of major downstream cytokine-receptor signaling pathways. (**A**) Sketch summarizing literature and own data [5] to illustrate complex downstream regulation of cytokine-receptor signaling cascade via MAP/ERK kinase (MEK1), phosphatidylinositol 3-kinase (PI3K) and c-Jun N-terminal kinase (JNK); e.g. the cytokine IL-6 is upregulated and secreted by palmitate in cultured detrusor myocytes (Fig. S2); (**B**–**D**) Cell proliferation, mitochondrial enzyme activity and antioxidant capacity of hBSMC after 48 h stimulation with 0.25 mM palmitate and inhibition of MEK1 pathway with PD98059 (**B**), PI3K pathway with LY294002 (**C**) and of JNK pathway with SP600125 (**D**). Data are shown as mean and SEM from three different cultures. Significant differences are indicated by bars. For appropriate medium controls refer to Fig.1A (48h stimulation).

Downstream of pSTAT3, the IL-6-JAK/STAT pathway can be regulated by MEK 1, PI3K and JNK signaling (Fig. 5A). Inhibition of MEK1 by PD98059 during stimulation with 0.25 mM palmitate had no influence on cell proliferation or antioxidant capacity but decreased mitochondrial enzyme activity (Fig. 5B). However, COX1 and PGC-1α, both involved in mitochondrial enzyme activity, were down regulated by inhibition of MEK1 pathway (Fig. 2B).

A more prominent regulation of cellular response was observed when phosphoinositol (PI)-3-kinase signaling was inhibited (LY294002). Palmitate reduced cell proliferation was dramatically further inhibited when palmitate was applied together the PI3K inhibitor LY294002 (Fig. 5C). Mitochondrial enzyme activity, which was enhanced by palmitate alone, was further enhanced when PI3K pathway was inhibited (Fig. 5C). Inhibition of PI3K pathway did not affect palmitate-induced augmentation of antioxidant capacity (Fig. 5C).

On gene expression level inhibition of PI3K during palmitate stimulation showed varying effects on the examined genes (Fig. 2). We found a down-regulation of 4E-BP1 and COX1 and up-regulation of PGC-1α gene expression levels when PI3K was inhibited (Fig. 2A and 2B).

Another major downstream pathway of cytokine signaling is characterized by its key-enzymes c-Jun N-terminal kinases (JNKs; Fig. 5A). Selective inhibition of JNK by SP600125 at 0.25 mM palmitate stimulation revealed further decrease in cell proliferation (Fig. 5D), while neither mitochondrial enzyme activity nor antioxidant capacity were affected significantly (Fig. 5D). Gene analysis showed enhanced BCL-XL (Fig. 2A) but decreased PGC-1α expression (Fig. 2B).

## Discussion

In the present study we were interested in the effect of palmitate on various cellular functions, on gene expression related to vital cell properties, and the involvement of the major cytokine signaling pathways into palmitate stimulated effects. We investigated the modulation of the cytokine receptor signaling cascade always related to palmitate stimulation and therefore we did not evaluate the effects of inhibitors alone on naive cells, which may be some limitation of the study regarding the analysis of cytokine receptor induced signaling cascades proper.

### Palmitate Influences Cell Vitality

Several studies have consistently demonstrated that elevated levels of plasma FFA, such as palmitate, were found in obesity related disorders [30]. Yet, it is unclear whether alterations in circulation palmitate contribute to elevated urinary tract infections [31]. Compromised voiding function was recently reported in a rat obesity model. Since induction of diabetes type 2 in those rats did not show significant differences to obesity alone, chronic obesity proper may be responsible for the bladder and urethral sphincter alterations [32]. However, plasma levels of FFA have not been addressed in this study. Both, increased risk of abacterial bladder inflammation and tissue dystrophy could be assigned to palmitate mediated downregulation of cell proliferation and upregulation of apoptosis. The primary aim of our study was to investigate the effect of palmitate on cell proliferation, mitochondrial enzyme activity and antioxidant capacity and secondly to analyze involved cytokine signaling key pathways.

### Inhibition of Cell Proliferation

Palmitate inhibits cell proliferation in hBSMC in time dependent manner (Fig. 1A) and simultaneous inhibition of NF-κB release using MG132 led to dramatic further downregulation of cell proliferation (Fig. 1C). It is well known, that NF-κB plays a fundamental role in cell survival and proliferation [33]. Our previous study showed significantly enhanced translocation of phosphorylated NF-κB into the nucleus of hBSMC when treated with palmitate [5]. Therefore we conclude that downregulation of cell proliferation occures despite of enhanced nuclear translocation of pNF-κB. However, palmitate can induce cytokine expression via NF-κB signaling as shown previously, revealing the significance of NF-κB for palmitate signaling in hBSMC [5].

Besides TLR4 mediated NF-κB release (Fig. 5A), NF-κB can also be activated through (PI3K) and its major downstream kinase, AKT [34]. To evaluate the influence of PI3K we inhibited PI3K with LY294002 and found a further significant decrease in cell proliferation (Fig. 5C). Furthermore we found that PI3K/mTOR regulated 4E-BP1, which in turn controls the initiation of translation of distinct classes of mRNAs [35], after palmitate stimulation (Fig. 2A).

Inhibition of NF-κB by the proteasome inhibitor MG132 enhanced these effects (Fig. 1C, Fig. 2A), indicating a central role of NF-κB in controlling hBSMC growth and vital functions. Interestingly, 4E-BP1 is able to inhibit cell cycle progression by selectively inhibiting the translation of messenger RNAs that encode proliferation promoting proteins [36].

Reduced cell proliferation might influence the capability of tissue remodeling and regeneration following insults to the urinary bladder. However, up to date data on morphological changes in detrusor smooth muscle cells in overactive bladder and other bladder dysfunctions are sparse. Most studies indicate alterations of basic smooth muscle cell functions on a cellular basis, i.e. cell-cell communication [10], cellular hypertrophy [37], and contractility [38]. However, proliferative capacity of smooth muscle cells and/or of interstitial cells might be crucial for remodeling of the bladder wall and diminishing this capacity might impair the ability of the bladder to functional regenerate following inflammation mediated insults.

### Palmitate Mediated Regulation of Mitochondria and Antioxidant Activity

In addition, our studies show that elevated palmitate levels lead to increased mitochondrial enzyme activity and antioxidant capacity in hBSMC (Fig. 1A). Indeed, obesity related disorders are linked to mitochondrial dysfunction [39] and palmitate is a potent trigger of those effects [40].

We here provide evidence that palmitate modulates protein carbonylation and lipid peroxidation (Fig. 1B). Palmitate mediated induction of ROS would lead to lipid peroxidation and protein carbonylation which is counteracted by upregulation of antioxidant capacity as a cellular protection mechanism. In hBSMC enhanced antioxidant capacity is not sufficient for successful prevention of lipid peroxidation but significantly reduces protein carbonylation. The Literature supports evidence that carbonylation of proteins such as actin, a protein involved in cell contractility of hBSMC, leads to drastic functional impairments [38]. Thus, in obese patients elevated palmitate may enhance functionality of certain proteins, e.g. smooth muscle cell actin and/or myosin, which in turn show higher calcium sensitivity [41] and may thereby contribute to bladder overactivity as seen associated with type II diabetes. This putative mechanism of palmitate-mediated effects might hold true even in other cellular systems.

Lipid peroxidation, which is known to impair lipid function, is another major direct effect of ROS and enhanced antioxidant capacity should prevent lipid peroxidation. However, we found increased staining for malone dialdehyde (MDA, Fig. 1B) following 48 h of stimulation with physiological concentration of palmitate, indicating membrane lipid peroxidation. This might well be due to the slow lipid turnover of several days [42] compared to the fast protein turnover rates usually within minutes to hours [43]. Due to this difference in half-life peroxidated lipids might be still detectable in membranes after 48 h.

For the first time our study reveals important insights, that elevated levels of FFAs cause the increased expression of peroxisome proliferator–activated receptor (PPAR –coactivator-1 (PGC-1) (Fig. 2B), a transcriptional activator that promotes oxidative capacity. Several researchers have reported that insulin-resistant states are characterized by a reduction in skeletal muscle of PGC-1α, the transcriptional coactivator driving the expression of many genes coding for proteins in mitochondria [40]. Thus, overexpression of PGC-1α in cultured myoblasts increases mitochondrial biogenesis and oxidative respiration [44]. The gene regulation of PGC-1α in hBSMC after palmitate induction (Fig. 2B) might be partially responsible for the increased mitochondrial activity (Fig. 1A).

Multiple intracellular pathways could be involved in the regulation of proliferation, apoptosis induction, mitochondrial activity and increase of antioxidant capacity. When blocking NF-κB signaling by inhibition of proteasome degradation of phosphorylated IkB-P during palmitate stimulation cell proliferation and antioxidant capacity was suppressed, while mitochondrial activity was not affected (Fig. 1c, Fig. 2). However, we found significant inhibition of gene expression in several genes again demonstrating the independence of gene regulation and protein expression [45].

### Palmitate Stimulates Cytokine Production and Release

In our previous studies, the free fatty acid palmitate induced time and concentration dependent IL-6 release in hBSMC, followed by autocrine and paracrine regulation of the IL6 pathway [5]. In the present study we provide further evidence for the involvement of JAK/STAT cytokine receptor pathway in palmitate-mediated effects (Fig. 3).

Studies investigating cytokine signaling in human bladder smooth muscle cells are rare; most studies have investigated cytokine induced IL-6 release by hBSMC [12]. To our best knowledge this is the first study targeting the downstream cytokine receptor signaling pathways of MEK1, PI3K and JNK and regulated genes involved in cell proliferation. These, the major pathways can be activated by a large variety of other factors, including growth factors [46], chemokines [7], [12], and survival factors [19].

Previously, we could show IL-6 release after palmitate stimulation (Fig. S2). The IL-6 induced autocrine and paracrine activation of JAK/STAT3 pathway results in intracellular signaling starting with phosphorylation of STAT3 (pSTAT3) [19]. Inhibition of NF-kB by MG 132 can downregulate this autocrine signaling indirectly (Fig. S3).

Direct cytokine signaling can be analyzed either by inhibition of the JAK/STAT3 inhibitor SOCS3 [47] (Fig. 4) leading to increase of phosphorylated STAT3 translocation into the nucleus (Fig. S3) [5] or by inhibition of the JAK/STAT3 complex via AG490 (Fig. 3B) [48]. Blocking SOCS3 expression by SOCS3 siRNA followed by 48 h of simulation with 0.25 mM palmitate significantly increased cell proliferation and augmented bladder smooth muscle antioxidant capacity (Fig. 2B). Consequently, the inhibition of the JAK/STAT pathway by AG490 caused down-regulation of cell proliferation (Fig. 3A) as described in various other studies [48], [49].

### Palmitate Related Cytokine Receptor Downstream Pathways

To identify the regulation of cell proliferation downstream JAK/STAT we inhibited MEK1 by PD98059, JNK by SP600125 and PI3K by LY294002 (Fig. 5). Both PI3K and JNK seem to be responsible for the observed down-regulation of cell proliferation by inhibition of JAK/STAT pathway by AG490.

In our experiments the eukaryotic translation initiation factor 4E-binding protein 1 (4E-BP1) was down-regulated by inhibition of PI3K (Fig. 2A). PI3K/AKT provides an indirect negative control of 4E-BP1 phosphorylation by inhibition of mTOR activation, leading to increased protein synthesis by decreased 4E-BP1 binding to the translation initiation site [36]. 4E-BP1 regulates proteins involved in cell proliferation and cell cycle progression and 4E-BP1 downregulation may therefore account for the observed decrease in cell proliferation [36].

Furthermore, inhibition of JNK leads to increased BCL-XL gene transcription (Fig. 2A) a gene, which inhibits cytochrome c release and downstream caspase 9 related apoptosis [50]. Palmitate is able to induce apoptosis [51]. Other authors provide compelling evidence that the JNK pathway is responsible for BCL-2 phosphorylation and therefore can directly regulate the cell proliferation [52]. We found no mRNA regulation of BCL-2 (data not shown) by palmitate stimulation or by inhibition of cytokine related pathways. Under all these conditions, inhibiting of JAK/STAT3, PI3K and JNK decreased cell proliferation (Fig. 5C and D).

Since those pathways are especially activated by IL-6, it seems that this cytokine may have an overall beneficial effect in case of cell deterioration. Other cytokines may, however, show divergent effects, depending on the degree of their activation of certain downstream pathways.

### Conclusions

Our study indicates that high levels of the saturated free fatty acid palmitate induce down regulation of cell proliferation through NF-κB and the formation of reactive intermediates leading to programmed cell death. Fatty acid-induced mitochondrial and antioxidant activity may contribute to human bladder disorders related to obesity. Additionally, palmitate-mediated production of ROS may cause significant cellular dysfunction that could contribute to the pathogenesis of those diseases without massive tissue reorganization due to programmed cell death. Cytokine signaling is involved in the process of cell proliferation and regulation of genes related to mitochondrial and antioxidant activity. Our findings suggest that metabolic and endocrine status is an important factor for the pathogenesis of bladder dysfunction. Pharmacologic approaches targeting and aiming to restore cytokine network in the bladder could be a novel therapeutic option.

## Supporting Information

Figure S1 **Palmitate effect on cell proliferation.** Phase contrast images of cultured hBSMC after 24 h and 48 h stimulation with 0.25 mM palmitate. Inset represents 2 fold magnification showing cellular alterations by 48 h stimulation with 0.25 mM palmitate: membrane blabbing (arrows) and detaching of cells (arrowheads).
(TIF)

Figure S2 **Alteration in IL-6 secretion of hBSMC.** Cultured hBSMC were pre-incubated 1 h with cytokine signaling pathway inhibitors (MG132, AG490, PD98059, LY294002 and SP600125) and following stimulated 48 h with 0.25 mM palmitate (black column). IL-6 secretion was measured using ELISA and data are shown as mean + SEM from three different cultures. Significant differences related to palmitate are indicated by bars.
(TIF)

Figure S3 **Palmitate influence pSTAT3 expression.** Confocal immunofluorescence of pSTAT3 (green) in cultured hBSMC after 48 h stimulation with 0.25 mM palmitate and palmitate plus NF-κB inhibitor MG132. Nuclei were stained with DAPI (blue). The inset represents negative staining control without using primary antibody against pSTAT3.
(TIF)

## References

[1] McGrother CW, Donaldson MM, Hayward T, Matthews R, Dallosso HM (2006). Urinary storage symptoms and comorbidities: a prospective population cohort study in middle-aged and older women.. Age Ageing.

[2] Bahillo-Curieses MP, Hermoso-Lopez F, Martinez-Sopena MJ, Cobreros-Garcia P, Garcia-Saseta P (2012). Prevalence of insulin resistance and impaired glucose tolerance in a sample of obese spanish children and adolescents.. Endocrine.

[3] LaRosa C, Meyers K (2010). Epidemiology of hypertension in children and adolescents.. J Med Liban.

[4] Sabin MA, De Hora M, Holly JM, Hunt LP, Ford AL (2007). Fasting nonesterified fatty acid profiles in childhood and their relationship with adiposity, insulin sensitivity, and lipid levels.. Pediatrics.

[5] Oberbach A, Schlichting N, Bluher M, Kovacs P, Till H (2010). Palmitate induced IL-6 and MCP-1 expression in human bladder smooth muscle cells provides a link between diabetes and urinary tract infections.. PLoS One.

[6] Benfield T, Jensen JS, Nordestgaard BG (2007). Influence of diabetes and hyperglycaemia on infectious disease hospitalisation and outcome.. Diabetologia.

[7] Staiger H, Staiger K, Stefan N, Wahl HG, Machicao F (2004). Palmitate-induced interleukin-6 expression in human coronary artery endothelial cells.. Diabetes.

[8] Bruce CR, Dyck DJ (2004). Cytokine regulation of skeletal muscle fatty acid metabolism: effect of interleukin-6 and tumor necrosis factor-alpha.. Am J Physiol Endocrinol Metab.

[9] Craig R, Larkin A, Mingo AM, Thuerauf DJ, Andrews C (2000). p38 MAPK and NF-kappa B collaborate to induce interleukin-6 gene expression and release. Evidence for a cytoprotective autocrine signaling pathway in a cardiac myocyte model system.. J Biol Chem.

[10] Heinrich M, Oberbach A, Schlichting N, Stolzenburg JU, Neuhaus J (2011). Cytokine effects on gap junction communication and connexin expression in human bladder smooth muscle cells and suburothelial myofibroblasts.. PLoS ONE.

[11] Neuhaus J, Heinrich M, Schwalenberg T, Stolzenburg JU (2009). TGF-beta1 Inhibits Cx43 Expression and Formation of Functional Syncytia in Cultured Smooth Muscle Cells from Human Detrusor.. Eur Urol.

[12] Bouchelouche K, Alvarez S, Horn T, Nordling J, Bouchelouche P (2006). Human detrusor smooth muscle cells release interleukin-6, interleukin-8, and RANTES in response to proinflammatory cytokines interleukin-1beta and tumor necrosis factor-alpha.. Urology.

[13] Tyagi P, Barclay D, Zamora R, Yoshimura N, Peters K (2010). Urine cytokines suggest an inflammatory response in the overactive bladder: a pilot study.. Int Urol Nephrol.

[14] Erickson DR, Xie SX, Bhavanandan VP, Wheeler MA, Hurst RE (2002). A comparison of multiple urine markers for interstitial cystitis.. J Urol.

[15] Neuhaus J, Schlichting N, Oberbach A, Stolzenburg JU (2007). [Lipopolysaccharide-mediated regulation of interleukin-6 in cultured human detrusor smooth muscle cells.].. Urologe A.

[16] Wang Z, Jin H, Xu R, Mei Q, Fan D (2009). Triptolide downregulates Rac1 and the JAK/STAT3 pathway and inhibits colitis-related colon cancer progression.. Exp Mol Med.

[17] Fukada T, Hibi M, Yamanaka Y, Takahashi-Tezuka M, Fujitani Y (1996). Two signals are necessary for cell proliferation induced by a cytokine receptor gp130: involvement of STAT3 in anti-apoptosis.. Immunity.

[18] Sasaki A, Yasukawa H, Suzuki A, Kamizono S, Syoda T (1999). Cytokine-inducible SH2 protein-3 (CIS3/SOCS3) inhibits Janus tyrosine kinase by binding through the N-terminal kinase inhibitory region as well as SH2 domain.. Genes Cells.

[19] Bode JG, Nimmesgern A, Schmitz J, Schaper F, Schmitt M (1999). LPS and TNFalpha induce SOCS3 mRNA and inhibit IL-6-induced activation of STAT3 in macrophages.. FEBS Lett.

[20] Baumann P, Mandl-Weber S, Oduncu F, Schmidmaier R (2009). The novel orally bioavailable inhibitor of phosphoinositol-3-kinase and mammalian target of rapamycin, NVP-BEZ235, inhibits growth and proliferation in multiple myeloma.. Exp Cell Res.

[21] Huang XF, Chen JZ (2009). Obesity, the PI3K/Akt signal pathway and colon cancer.. Obes Rev.

[22] Zheng CF, Guan KL (1993). Properties of MEKs, the kinases that phosphorylate and activate the extracellular signal-regulated kinases.. J Biol Chem.

[23] Chang MC, Wu HL, Lee JJ, Lee PH, Chang HH (2004). The induction of prostaglandin E2 production, interleukin-6 production, cell cycle arrest, and cytotoxicity in primary oral keratinocytes and KB cancer cells by areca nut ingredients is differentially regulated by MEK/ERK activation.. J Biol Chem.

[24] Rahman I (2003). Oxidative stress, chromatin remodeling and gene transcription in inflammation and chronic lung diseases.. J Biochem Mol Biol.

[25] Weigert C, Brodbeck K, Staiger H, Kausch C, Machicao F (2004). Palmitate, but not unsaturated fatty acids, induces the expression of interleukin-6 in human myotubes through proteasome-dependent activation of nuclear factor-kappaB.. J Biol Chem.

[26] Alessi DR, Cuenda A, Cohen P, Dudley DT, Saltiel AR (1995). PD 098059 is a specific inhibitor of the activation of mitogen-activated protein kinase kinase in vitro and in vivo.. J Biol Chem.

[27] Tao QS, Huang HL, Chai Y, Luo X, Zhang XL (2012). Interleukin-6 up-regulates the expression of interleukin-15 is associated with MAPKs and PI3-K signaling pathways in the human keratinocyte cell line, HaCaT.. Mol Biol Rep.

[28] Bennett BL, Sasaki DT, Murray BW, O’Leary EC, Sakata ST (2001). SP600125, an anthrapyrazolone inhibitor of Jun N-terminal kinase.. Proc Natl Acad Sci U S A.

[29] Lee DH, Goldberg AL (1998). Proteasome inhibitors: valuable new tools for cell biologists.. Trends Cell Biol.

[30] Wilding JP (2007). The importance of free fatty acids in the development of Type 2 diabetes.. Diabet Med.

[31] Stapleton A (2002). Urinary tract infections in patients with diabetes.. Am J Med.

[32] Gasbarro G, Lin DL, Vurbic D, Quisno A, Kinley B (2010). Voiding function in obese and type 2 diabetic female rats.. Am J Physiol Renal Physiol.

[33] Muller M, Morotti A, Ponzetto C (2002). Activation of NF-kappaB is essential for hepatocyte growth factor-mediated proliferation and tubulogenesis.. Mol Cell Biol.

[34] Harvey RD, Lonial S (2007). PI3 kinase/AKT pathway as a therapeutic target in multiple myeloma.. Future Oncol.

[35] Burnett PE, Barrow RK, Cohen NA, Snyder SH, Sabatini DM (1998). RAFT1 phosphorylation of the translational regulators p70 S6 kinase and 4E-BP1.. Proc Natl Acad Sci U S A.

[36] Hay N, Sonenberg N (2004). Upstream and downstream of mTOR.. Genes Dev.

[37] Chang S, Hypolite JA, Mohanan S, Zderic SA, Wein AJ (2009). Alteration of the PKC-mediated signaling pathway for smooth muscle contraction in obstruction-induced hypertrophy of the urinary bladder.. Lab Invest.

[38] Dalle-Donne I, Rossi R, Giustarini D, Gagliano N, Lusini L (2001). Actin carbonylation: from a simple marker of protein oxidation to relevant signs of severe functional impairment.. Free Radic Biol Med.

[39] Bournat JC, Brown CW (2010). Mitochondrial dysfunction in obesity.. Curr Opin Endocrinol Diabetes Obes.

[40] Coll T, Jove M, Rodriguez-Calvo R, Eyre E, Palomer X (2006). Palmitate-mediated downregulation of peroxisome proliferator-activated receptor-gamma coactivator 1alpha in skeletal muscle cells involves MEK1/2 and nuclear factor-kappaB activation.. Diabetes.

[41] Shao CH, Rozanski GJ, Nagai R, Stockdale FE, Patel KP (2010). Carbonylation of myosin heavy chains in rat heart during diabetes.. Biochem Pharmacol.

[42] van Meer G, Voelker DR, Feigenson GW (2008). Membrane lipids: where they are and how they behave.. Nat Rev Mol Cell Biol.

[43] Eden E, Geva-Zatorsky N, Issaeva I, Cohen A, Dekel E (2011). Proteome half-life dynamics in living human cells.. Science.

[44] Wu Z, Puigserver P, Andersson U, Zhang C, Adelmant G (1999). Mechanisms controlling mitochondrial biogenesis and respiration through the thermogenic coactivator PGC-1.. Cell.

[45] Varambally S, Yu J, Laxman B, Rhodes DR, Mehra R (2005). Integrative genomic and proteomic analysis of prostate cancer reveals signatures of metastatic progression.. Cancer Cell.

[46] Yu JH, Kim KH, Kim H (2008). SOCS 3 and PPAR-gamma ligands inhibit the expression of IL-6 and TGF-beta1 by regulating JAK2/STAT3 signaling in pancreas.. Int J Biochem Cell Biol.

[47] Johnston JA, O’Shea JJ (2003). Matching SOCS with function.. Nat Immunol.

[48] Watanabe S, Mu W, Kahn A, Jing N, Li JH (2004). Role of JAK/STAT pathway in IL-6-induced activation of vascular smooth muscle cells.. Am J Nephrol.

[49] Seo IA, Lee HK, Shin YK, Lee SH, Seo SY (2009). Janus Kinase 2 Inhibitor AG490 Inhibits the STAT3 Signaling Pathway by Suppressing Protein Translation of gp130.. Korean J Physiol Pharmacol.

[50] Li P, Nijhawan D, Budihardjo I, Srinivasula SM, Ahmad M (1997). Cytochrome c and dATP-dependent formation of Apaf-1/caspase-9 complex initiates an apoptotic protease cascade.. Cell.

[51] Kong JY, Rabkin SW (2000). Palmitate-induced apoptosis in cardiomyocytes is mediated through alterations in mitochondria: prevention by cyclosporin A. Biochim Biophys Acta.

[52] Heo SK, Yun HJ, Park WH, Park SD (2008). Emodin inhibits TNF-alpha-induced human aortic smooth-muscle cell proliferation via caspase- and mitochondrial-dependent apoptosis.. J Cell Biochem.

