# Supplementary figures and images for: Free Fatty Acid Palmitate Impairs the Vitality and Function of Cultured Human Bladder Smooth Muscle Cells
Source: PLoS One. 2012 Jul 13;7(7):e41026. doi: 10.1371/journal.pone.0041026 (PMC3396599; doi:10.1371/journal.pone.0041026)

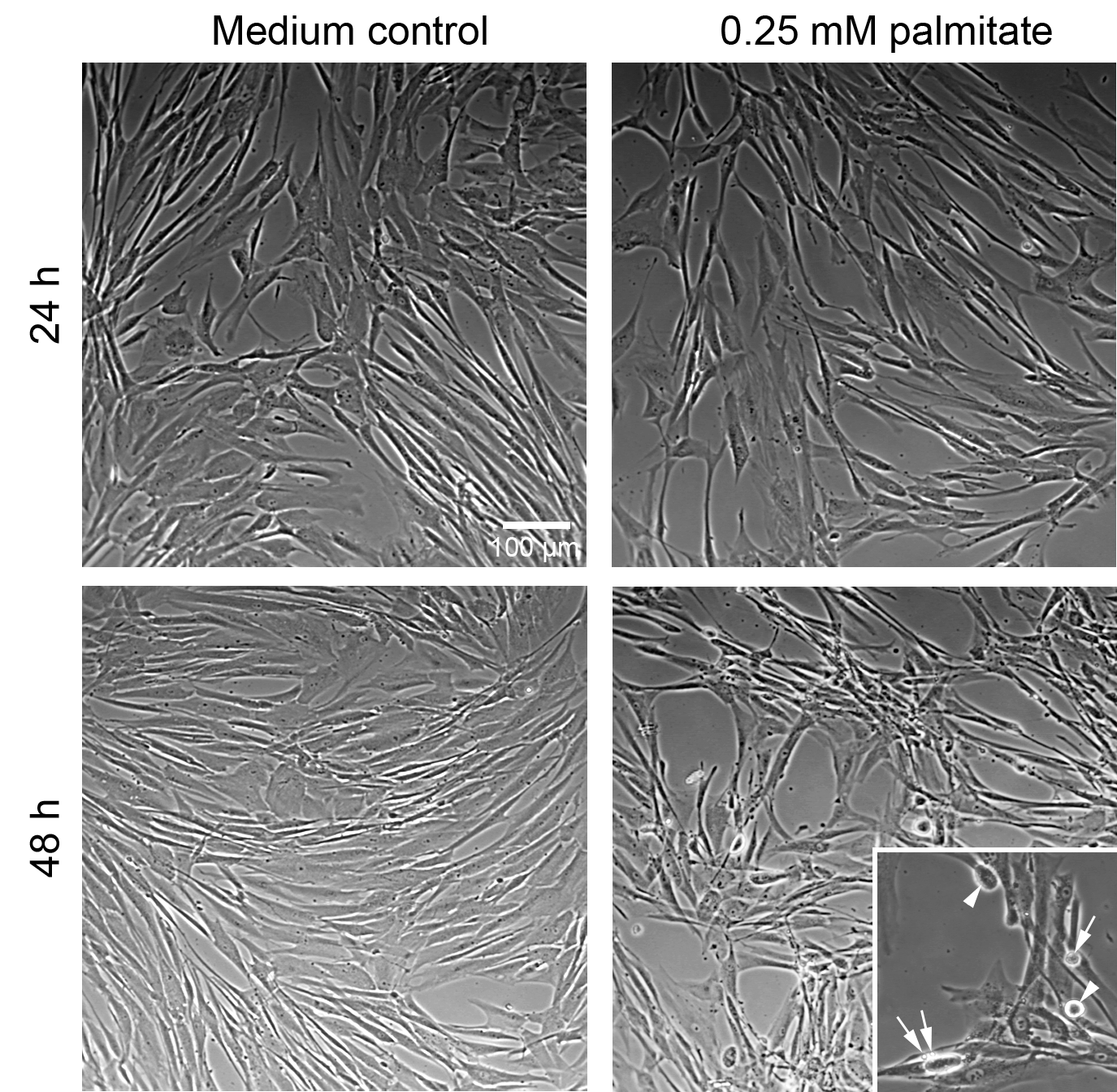

Supplement: Figure S1 — Palmitate effect on cell proliferation. Phase contrast images of cultured hBSMC after 24 h and 48 h stimulation with 0.25 mM palmitate. Inset represents 2 fold magnification showing cellular alterations by 48 h stimulation with 0.25 mM palmitate: membrane blabbing (arrows) and detaching of cells (arrowheads). (TIF) [file pone.0041026.s001.tif]

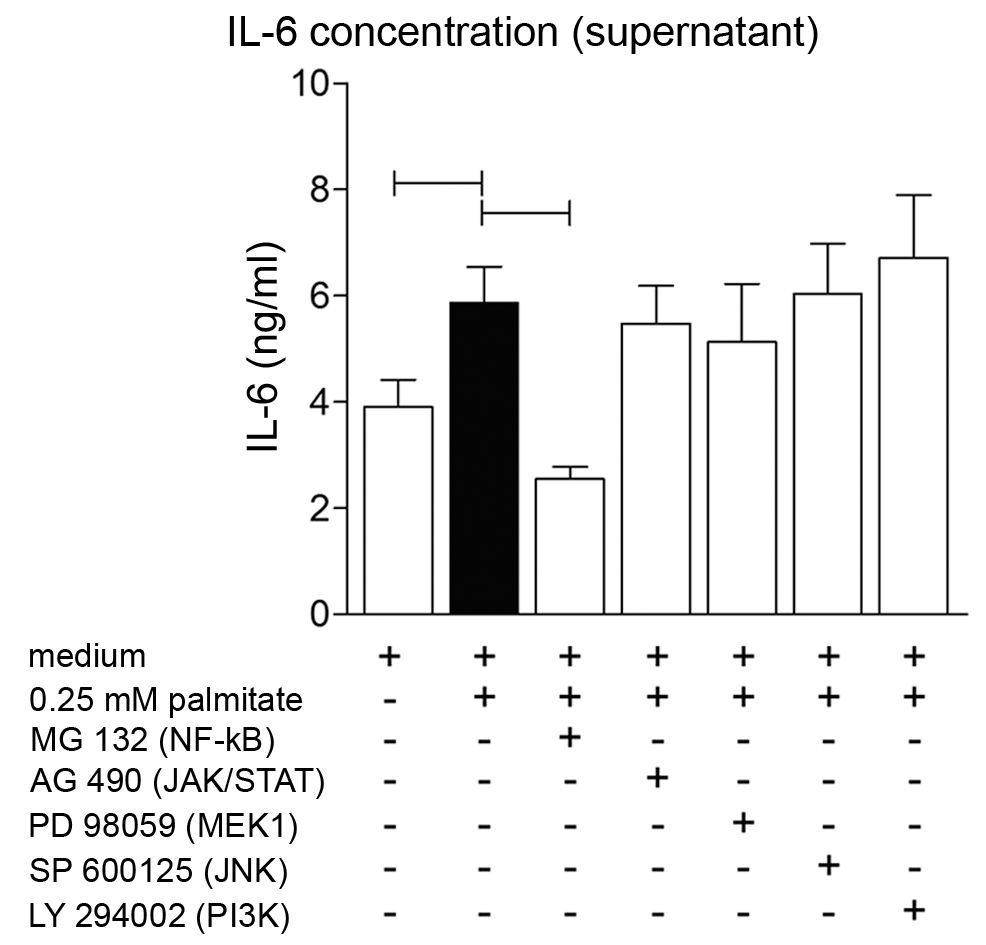

Supplement: Figure S2 — Alteration in IL-6 secretion of hBSMC. Cultured hBSMC were pre-incubated 1 h with cytokine signaling pathway inhibitors (MG132, AG490, PD98059, LY294002 and SP600125) and following stimulated 48 h with 0.25 mM palmitate (black column). IL-6 secretion was measured using ELISA and data are shown as mean + SEM from three different cultures. Significant differences related to palmitate are indicated by bars. (TIF) [file pone.0041026.s002.tif]

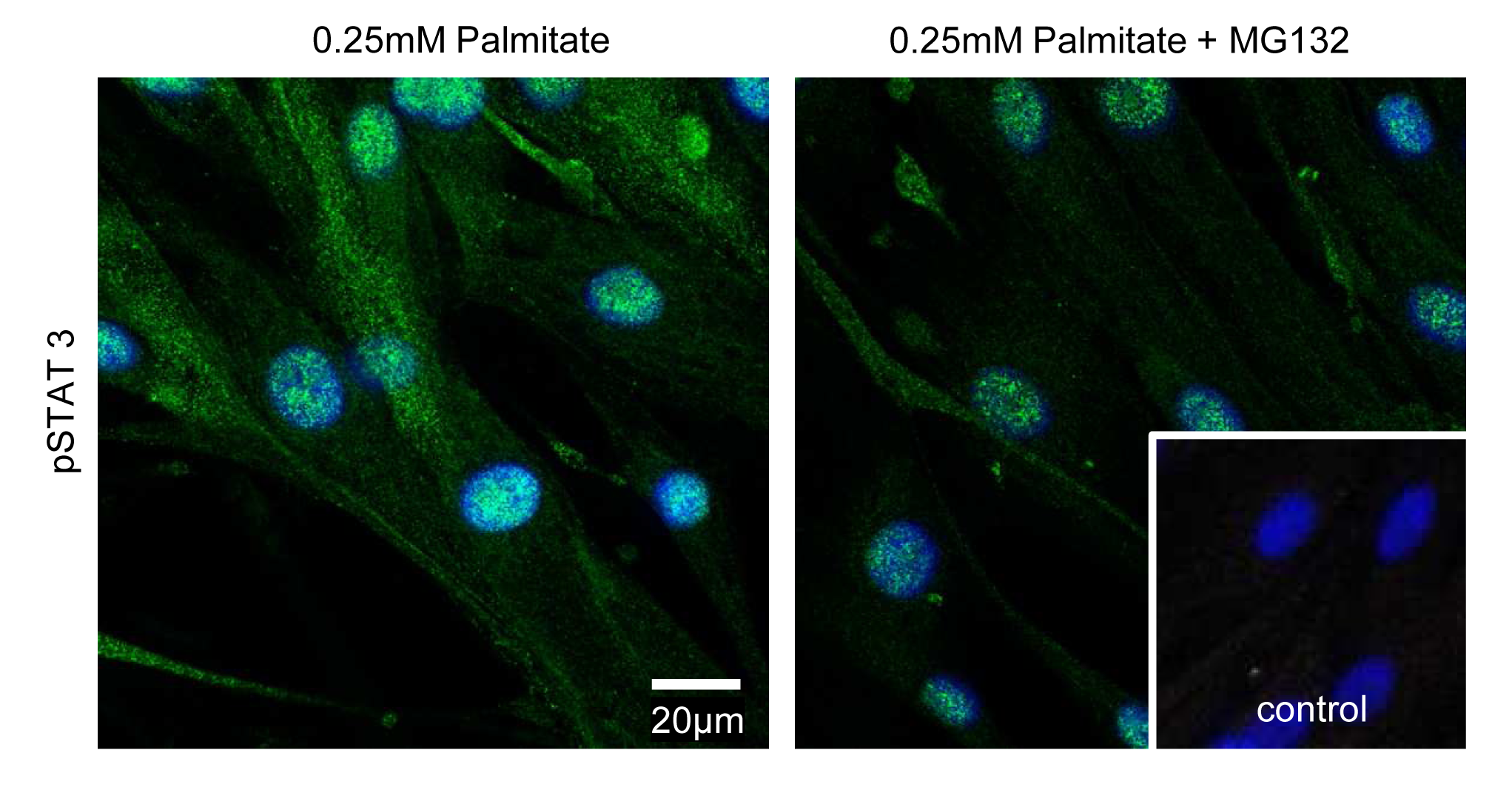

Supplement: Figure S3 — Palmitate influence pSTAT3 expression. Confocal immunofluorescence of pSTAT3 (green) in cultured hBSMC after 48 h stimulation with 0.25 mM palmitate and palmitate plus NF-κB inhibitor MG132. Nuclei were stained with DAPI (blue). The inset represents negative staining control without using primary antibody against pSTAT3. (TIF) [file pone.0041026.s003.tif]
